# The Multi-Gas Sensor for Remote UAV and UGV Missions—Development and Tests

**DOI:** 10.3390/s21227608

**Published:** 2021-11-16

**Authors:** Miron Kaliszewski, Maksymilian Włodarski, Jarosław Młyńczak, Bartłomiej Jankiewicz, Lukas Auer, Bartosz Bartosewicz, Malwina Liszewska, Bogusław Budner, Mateusz Szala, Bernhard Schneider, Günter Povoden, Krzysztof Kopczyński

**Affiliations:** 1 Institute of Optoelectronics, Military University of Technology, ul. gen. Sylwestra Kaliskiego 2, 00-908 Warsaw, Poland; maksymilian.wlodarski@wat.edu.pl (M.W.); jaroslaw.mlynczak@wat.edu.pl (J.M.); bartlomiej.jankiewicz@wat.edu.pl (B.J.); bartosz.bartosewicz@wat.edu.pl (B.B.); malwina.liszewska@wat.edu.pl (M.L.); boguslaw.budner@wat.edu.pl (B.B.); krzysztof.kopczynski@wat.edu.pl (K.K.); 2 Airnail e.U—Wings for Things, Haslbergerweg 14, 5023 Salzburg, Austria; al@airnail.at; 3 Faculty of Advanced Technologies and Chemistry, Military University of Technology, ul. gen. Sylwestra Kaliskiego 2, 00-908 Warsaw, Poland; mateusz.szala@wat.edu.pl; 4 Department EWBT, Defence Technology Agency, Rossauer Laende 1, 1090 Vienna, Austria; bernhard.schneider@bmlv.gv.at; 5 Austrian Armed Forces CBRN Defence Centre, ABC-Abwehrzentrum, Str., 1090 Vienna, Austria; guenter.povoden@bmlv.gv.at

**Keywords:** electrochemical gas sensor, UGV, UAV, remote detection, downwash

## Abstract

In this article, we present a versatile gas detector that can operate on an unmanned aerial vehicle (UAV) or unmanned ground vehicle (UGV). The device has six electrochemical modules, which can be selected to measure specific gases, according to the mission requirements. The gas intake is realized by a miniaturized vacuum pump, which provides immediate gas distribution to the sensors and improves a fast response. The measurement data are sent wirelessly to the operator’s computer, which continuously stores results and presents them in real time. The 2 m tubing allows measurements to be taken in places that are not directly accessible to the UGV or the UAV. While UAVs significantly enhanced the versatility of sensing applications, point gas detection is challenging due to the downwash effect and gas dilution produced by the rotors. In our work, we demonstrated the method of downwash effect reduction at aerial point gas measurements by applying a long-distance probe, which was kept between the UAV and the examined object. Moreover, we developed a safety connection protecting the UAV and sensor in case of accidental jamming of the tubing inside the examined cavity. The methods presented provide an effective gas metering strategy using UAVs.

## 1. Introduction

The rapid detection and identification of chemical agents at the place of an incident is crucial for fast risk assessment and, as a result, for a reduction in the severity of the incident’s consequences, and an improvement in the protection of the responding personnel. Nowadays, detection and identification are very often carried out with the support of unmanned aerial vehicles (UAV) and unmanned ground vehicles (UGV), which allow the presence of human factors directly in the area of the hazard to be limited. The replacement of human factors by machines also has other benefits because the size and properties of the UAV and UGV platforms can be tailored depending on the mission requirements. The recent advances in battery performance, microelectronics, and miniaturized powerful electrical motor technology have especially contributed to the fast development and common availability of UAVs, which have become widely used in various areas, including the military, security, transport, observation, and air monitoring [1]. Therefore, it is not surprising that, nowadays, UAVs and/or UGVs play a critical role in several rescue or emergency scenarios, such as searching for victims in hard-to-reach terrain [2], remote bomb disposal [3], biological warfare agent detection [4], industrial installation leak inspections [5], and special equipment transportation [6].

First responders and researchers can benefit from a broad range of instrumentation that allows the detection and identification of solid, liquid, and gaseous chemical agents. However, commercially available chemical sensors, commonly used by first responders, are often unsuitable for use on unmanned platforms, especially UAVs, since they do not meet certain requirements, such as weight, size, shape, power supply, and dedicated connectors. Other disadvantages of off-the-shelf devices often include the lack of real-time and remote data transfer from the sensor to the operator who stays on the ground, or the lack of control of some useful functions via the manufacturer’s software [7]. Therefore, many commercial chemical sensors could be difficult to adapt to the specific system requirements. Advances in flexible and programable electronics, IoT technology, and the broad availability of high-quality environmental sensors have motivated multiple groups all over the world to develop unique smart sensors or solutions that are successfully used when commercial ones are unavailable. Such tailored sensing instruments can cover the fields of interest of specific research groups. An example of such a system was designed by Ahlawat et al. to study the gaseous concentration in the atmospheric boundary layer [8].

Some scientists even openly disseminate the results of their work on sensor construction to a broader audience, and provide a detailed description, which allows a low-cost environmental monitoring system to be built [9]. In recent years, research and development in the field of sensing technologies have resulted in the introduction of dedicated sensing technologies to the market of UAVs. Currently, there are at least a few commercial UAV-dedicated solutions, which are tailored for urban air quality and emission/odor monitoring, gas leakage recognition, and other applications related to chemical agent detection [10,11,12].

Several research studies have demonstrated the successful combination of UAVs and sensing technologies for visual inspection of buildings and construction conditions [13,14], outdoor air quality [15], and climatic research [16]. Different strategies have been applied, allowing sample collection for further laboratory analysis [16], or on-site sensing using detectors that allow data transfer to the operator. Particularly useful solutions allow high-resolution measurement, real-time data transfer, and spatiotemporal indexing. Such functionalities have been successfully used in various types of research, such as collecting information on gas emissions in volcano craters [17], odor source localization [18], gas leaks [19], and particulate matter distribution in the atmosphere [20,21].

Despite there being many studies and various approaches, there are still several problems related to the use of sensing technologies on unmanned platforms, especially on UAVs, which are yet to be solved; for example, while data that are recorded with UAV devices working in remote mode (e.g., cameras, lidars, and rangefinders) are unaffected, in situ sensors are strongly impacted by ambient air turbulence. In addition, proximity operations focused on point gas detection are challenging, and they run the risk of UAV and sensor damage. Furthermore, maneuvering the UAV in the vicinity of small cavities is complicated. A serious problem stems from the downwash effect related to vertical airflow disturbances, which has an impact on the measured gas concentration. Therefore, the exploration of new measurement approaches can improve the accuracy of measurements. Numerous strategies and sensor locations on the UAV have been investigated to obtain optimal sampling conditions [19]. Some of these investigations presented sensors with a sampling inlet mounted under [22] or on [23] the UAV’s hull. To diminish the propeller effect, extended stiff inlets, arranged above [16,24] or parallel [25] to the propeller plane, were also tested.

In this article, we present the results of our studies on the development of the ChemDet (chemical detector), a versatile multi-gas sensor for remote gas sensing using unmanned aerial/ground vehicles (UAV/UGV), with features that minimize or avoid common problems in the field of remote gas sensing. According to our knowledge, it is one of the first attempts allowing point gas sensing, minimizing the rotor air blast, and using a UAV hovering over the object. A similar probing solution was demonstrated in recently reported studies, by Burgués et al. The researchers used 10 m flexible tubing for gas sampling over wastewater treatment plants [19]. Our approach was developed independently, and it was focused on the point inspection of small cavities. For this reason, we also applied a specially shaped weight that facilitated inserting the probe to examine the object, and a protective connection allowing the emergency release of the UAV and sensor, in case of the tubing jamming in the cavity being examined. The flexible tubing has the following advantages over rigidly mounted pipes: (1) the rotor blast can be minimalized, since the vertical tubing length can be a few meters long; (2) the aircraft take-off is easier and more comfortable, due to less space being needed at the front of the UAV (horizontal pipe) or under the UAV (vertical pipe); (3) flexible tubing facilitates the UAV maneuvering for obstacle avoidance, as well as allowing bumping against the objects. The sensor described is the final version of the system, which has undergone several modifications and improvements based on the results of field trials.

## 2. Materials and Methods

### 2.1. Chemicals

The target gases and vapors, ammonia (NH_3_), nitric acid (HNO_3_), nitric oxides (NO*_x_*), hydrogen chloride (HCl), sulfuric acid (H_2_SO_4_), and hydrogen peroxide (H_2_O_2_), were measured from the above corresponding concentrated aqueous solutions of NH_3_, HNO_3_, HCl, H_2_SO_4_, and H_2_O_2_, respectively. The chemicals belong to the group of toxic industrial chemicals (TICs), while H_2_O_2_ is used in the production of homemade explosives. All chemicals were purchased from Sigma Aldrich (Poznań, Poland) and used as received. The chemicals selected for testing intentionally do not exactly match all the sensors used in the tests. In our studies, in addition to testing the functionality of the developed detector, we also wanted to test the cross-sensitivity of the sensors selected for our detector.

### 2.2. Instrument Description

The ChemDet (Institute of Optoelectronics, WAT, Warsaw, Poland) is a multi-gas sensor for use on unmanned platforms based on electrochemical gas sensing. The main components of ChemDet include a measurement chamber with six gas sensors, a sampling system (pump and gas intake system), a power source (Li-ion battery pack), and an electronic module for sensor control and data collection. The overall cost of a complete sensor is about EUR 1700, where the high-quality gas modules make up over 70% of the price.

**Figure 1:**
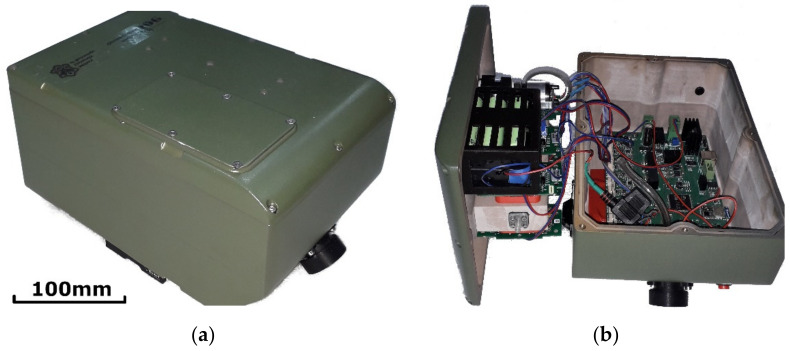
ChemDet sensor. (**a**) main view, (**b**) with open lid.

The sensor enclosure was made of lightweight composite material shaped with CNC milling and it was spray-painted with army green spray paint (Figure 1). The design had to meet specified requirements, which included the following: (a) sufficient room for the chamber with sensors, power supply, pump, and electronics; (b) possibly a flat box to hitch under the UAV; (c) compromise of the horizontal position of the sensor chamber and possible vertical tubing connection, obtained by bias at the bottom of the enclosure; (d) design of the enclosure to be watertight and gastight to avoid damage of the internal parts when operating in rain or a corrosive environment, the housing thus being a fold of two shells with a rubber seal.

The versatile design of the ChemDet makes it suitable for a wide range of operations and missions. It can operate as a handheld device carried by a person, “sniffing” ambient air at specific locations. Optionally, the ChemDet can operate on a UGV. In our solution, the sensor is attached to the UGV platform using NATO accessory rails mounted on the bottom of the enclosure. The NATO accessory rail is a proven and standardized mounting interface that can be tightened or loosened with a single hand movement allowing quick assembly or transfer of the ChemDet to a different platform. For UAV missions, the ChemDet can be installed under the UAV with cable ties slotted into dedicated cut-outs in the enclosure.

The ChemDet is turned on with one button that activates Wi-Fi, pump, and electronics with electrochemical modules. The booting procedure lasts about 60 s. The signals from the sensors are read every second and are sent to the computer via a 2.4 GHz Wi-Fi connection. The data are displayed on the receiver PC as timeline concentration graphs or as green concentration numbers. When the pre-set threshold (adjusted manually according to the mission requirements) is exceeded, the concentration number turns red. The sensor start-up procedure was simplified to a one-button start and the display program is designed to be possibly intuitive. The basic ChemDet parameters are presented in Table 1.

### 2.3. Measurement Chamber

**Figure 2:**
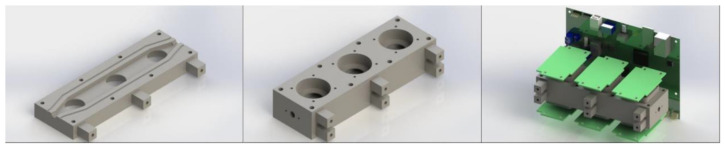
The 6-slotted sensor chamber layout. (**left**) lower part of the chamber; (**middle**) assembled chamber; (**right**) assembled chamber with attached sensors and circuit boards.

Up to six different gases can be continuously and simultaneously measured using electrochemical gas modules (Membrapor AG, Wallisellen, Switzerland), which are enclosed in a common flow chamber. The final version is based on a 6-slotted chamber (Figure 2). In such a design, the chamber volume was 6.8 cm^3^ and so the time necessary for aspired air to reach the sensing modules is negligible.

The sensors were selected to respond to highly hazardous toxic industrial chemicals (TICs), including Cl_2_, NH_3_, NO_2_, NO, and H_2_S. In addition, we included a H_2_O_2_ sensor for potential application in the detection of illicit homemade explosives, such as laboratory-manufactured triacetone triperoxide (TATP) or hexamethylene triperoxide diamine (HMTD). 

### 2.4. Operation Principle

The sensor configuration can be selected according to the mission requirements. Some individual experiments were conducted using different sensor sets, including those listed in Table 2. To improve the efficiency and detection rate, the air is continuously aspired and flows through the sensor chamber with the use of a miniaturized vacuum pump. The target gases or vapors induce an electrochemical reaction at the assigned sensor and, as a result, an electric signal, proportional to the gas concentration, is generated at the sensor’s electrodes. For proper operation, the sensors require a potentiostat circuit. This circuit can be configured to have the sensing and reference electrodes at the same potential (non-biased sensor) or to maintain these electrodes at a different voltages (biased sensor). The circuit sustains the potential difference at a stable level, characteristic to a specific sensor, by adjusting the potential of the counter electrode. Among the electrochemical modules used, the NO and H_2_O_2_ sensors required a biased configuration with a potential difference of 300 mV. The remaining sensors required the potential difference to be equal to 0 mV. The measurement range and basic parameters of the sensors are presented in Table 2.

According to the datasheets, the full response time of gas sensors ranges from about 10 to 60 s. This suggests that the concentration readings of the gases can be affected to a varying degree depending on sensor response time. To reduce the time lag, the sensors with a longer response time were placed closer to the gas inlet. The flow rate depended on the motor rotation speed, which can be controlled with the potentiometer on the electronic board and was set to 2.3 L/min. Assuming a given flow rate and 2 m long tubing (inner diameter 3 mm), the gas/vapor travels from tubing inlet to detector in ca. 0.7 s. This shows that sensor response time delay, due to sample transport along the tubing, is negligible.

### 2.5. The Gas Intake System

**Figure 3:**
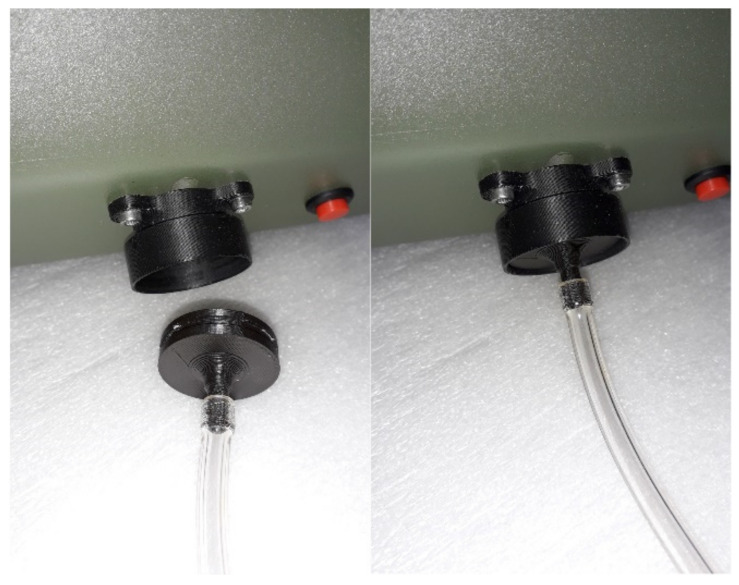
A magnet-based safety connection for the probing tube.

The ChemDet’s gas intake system allows either direct sensing of ambient air through the air inlet or through a 2 m long tubing (Tygon^®^ R-3603, Sigma-Aldrich, Poznań, Poland), which was exploited for gas detection inside waste bins or containers. The use of tubing has several advantages. Firstly, it allows avoidance of or reduction in the downwash effect when ChemDet is used on UAVs. In addition, the spindle-shaped weight on the tube tip facilitated the tubing placement and prevented its jamming inside the object being examined. However, accidental tubing jamming could damage the UAV and sensor. Therefore, for security reasons we have developed a magnet-based safety connection for the probing tube, which can be easily disconnected from ChemDet by pulling the UAV up (Figure 3).

## 3. Results and Discussion

### 3.1. Development of the ChemDet Sensor

During the development of the chemical sensor, several issues have to be considered, such as targeted chemicals, detection method, the way it is used (i.e., person, UGV, or UAV), technical requirements (i.e., weight, size, power supply, and communication), and price. Our goal was to develop a versatile and cheap chemical sensor for the remote detection of toxic chemicals.

### 3.2. Laboratory Tests of the Sensor

**Figure 4:**
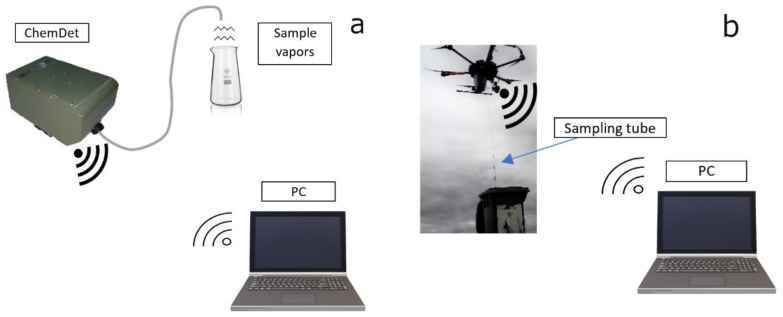
(**a**) Laboratory set up, (**b**) field tests.

**Figure 5:**
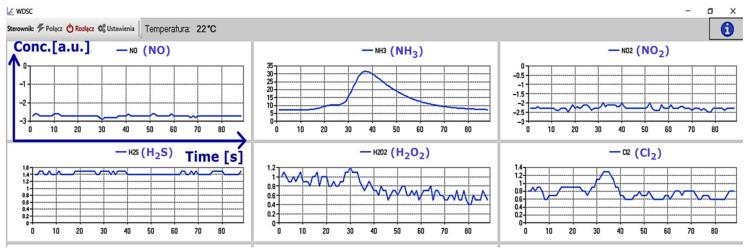
A screenshot presenting the results of the measurements of 25% ammonia in the laboratory. The axis labels and captions were inserted in the first plot for a clear presentation.

A schematic drawing of the laboratory setup is presented in Figure 4a. The tests were carried out at the Institute of Optoelectronics, at the Military University of Technology. We also investigated potential problems appearing during measurements, amongst other issues related to the sensors’ cross-sensitivity, which were apparent during tests, and were noted by the manufacturer in the specification sheets [26]. This problem has also been previously reported in the literature, and it is common for electrochemical sensors [27]. Ammonia and H_2_O_2_ were analyzed using vapors of the substances from their solutions in water. The responses of the sensors were also tested using gases and vapors from the above concentrated solutions of HCl, HNO_3_, and H_2_SO_4_. Since the project was focused on the detection of target gases or vapors, calibration of the sensor was not necessary. However, the software allows implementation of the calibration curve based, for example, on reference device readings. The sensor readings were compared as arbitrary units (a.u.) relating to the recorded electrical signal. Example of the data displayed during the measurement are presented in Figure 5.

**Figure 6:**
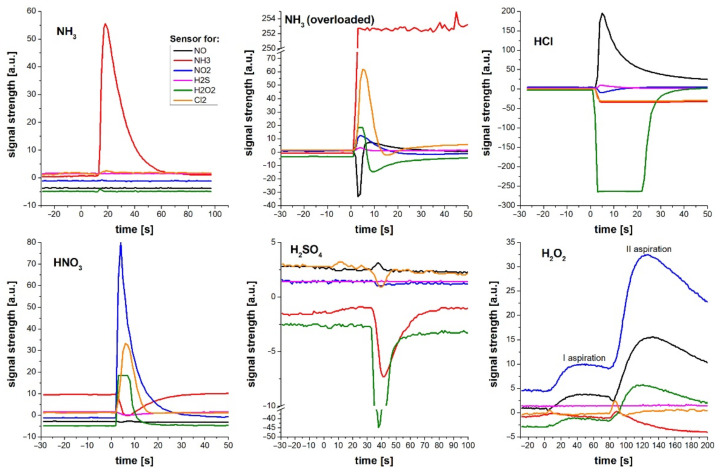
Specific sensor responses to different gases and vapors from above solutions of NH_3_ (in the range of measured concentration and saturation), HCl, HNO_3_, H_2_SO_4_, and H_2_O_2_. The I and II aspiration on the H_2_O_2_ plot refers to moving the aspirating tubing in and out of the place of gas emission. Some sensors show negative response that occurs for some gases and cannot be treated as a real physical result. Since sensors are calibrated to a specific redox reaction, another gas can produce reversed current on the electrodes.

The responses of specific sensors treated, at the same time, with a known gas are presented in Figure 6. The responses of various sensors to the target gas occurred at a similar time, so the results obtained were more uniform than the data declared by the manufacturer (Table 2). It should be noted that the gas concentration in the chamber changes dynamically. Therefore, it is likely that sensors with longer response times will produce underestimated signals, due to the longer time required for their maximum response. 

While the response time differences were not substantial, significant variability in the response patterns to various agents between the sensors was observed.

### 3.3. Sensor Field Tests

The sensor field tests were performed in the military training area in Allentsteig, Austria. Tests have been carried out in spring and autumn, in various environmental and weather conditions.

#### 3.3.1. UGV Chemical Detection

**Figure 7:**
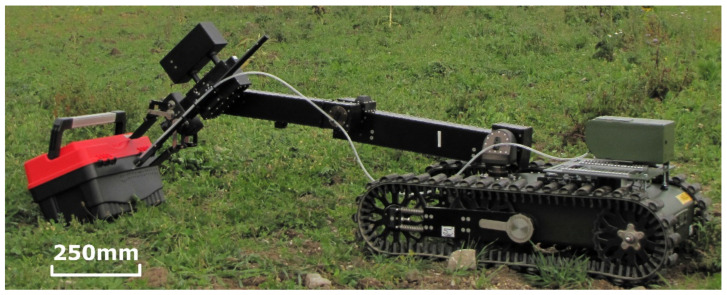
ChemDet mounted on the Taurob Tracker UGV platform.

The ChemDet was installed on the body of the UGV (Taurob Tracker GmbH, Vienna, Austria) [28] and the tubing was attached to the holder on the robot arm. The UGV operator maneuvered the UGV’s arm around the examined object to find the area where contamination could be detected. A photo of the UGV, with the ChemDet performing measurements, is presented in Figure 7. Due to the relatively steady-state conditions and cameras, a trained robot operator easily manipulates the robot arm to perform screening of different areas around the examined object. The main problem in volatile chemical detection stems from the weather conditions, especially on windy days. The use of the ChemDet with tubing allows the sensor to be placed on the body of the UGV, instead of its arm, which therefore, allows full arm and gripper functionality to be maintained. In addition, it prevents contamination of the sensor.

#### 3.3.2. Airborne Chemical Detection on UAVs

**Figure 8:**
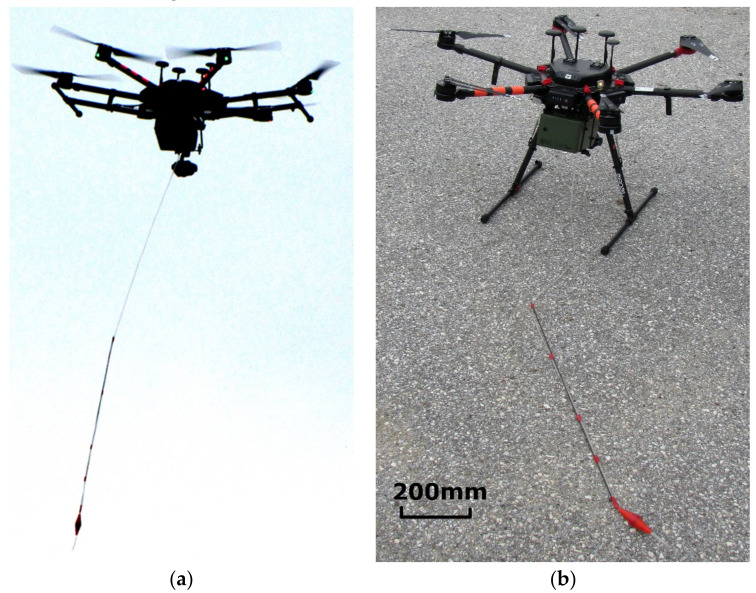
The UAV with ChemDet and tubing system (**a**) during flight and (**b**) before take-off.

During the project, we performed testing of the ChemDet detector on UAVs of several sizes and, consequently, different payloads. Different disturbing effects on the measurements were also observed. The first version of the ChemDet was tested on the UAV (DJI Inspire 2–not shown), while its final version was tested on the UAV (DJI M600) shown in Figure 8.

- For downwash testing, the DJI Inspire 2, Shenzhen, China was used [29].
- To carry the ChemDet sensor, we used multiple UAVs, but a DJI M600 is shown in Figure 8 [30].

The downwash problem is commonly known and has been widely discussed when the UAV–gas sensor combination has been studied. The rotating propellers produce strong and turbulent airflow under the aircraft, which can dilute or even push the air mass, thereby removing the target gas. Therefore, the downwash can significantly affect the sensor’s readings, due to the altered gas concentration below the UAV [1,31,32,33].

**Figure 9:**
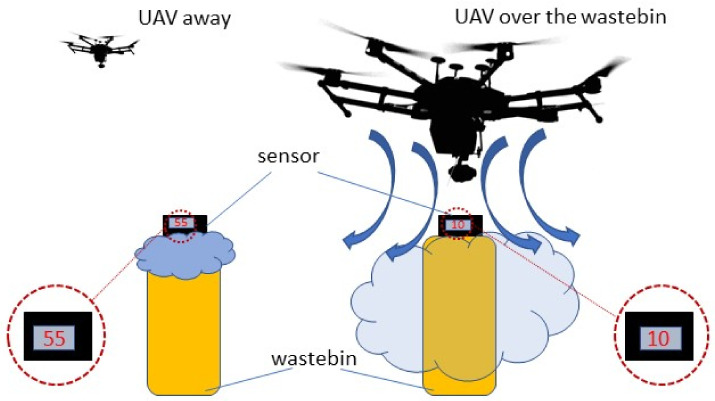
Experiment with ChemDet mounted on the wastebin, allowing the downwash effect to be measured.

To check the downwash effect, the ChemDet sensor was mounted on the wastebin, with the end of the sampling tube outside the wastebin, but close to its opening (Figure 9). During these measurements, we tested the influence of the air disturbance caused by the UAV on the signal level measured for NH_3_. The ammonia concentration was 2–5 times lower when the UAV was hovering over the wastebin, compared to the aircraft hovering away. Based on the results obtained, it can be concluded that the air disturbance caused by the UAV highly affects the readings of the ChemDet sensor placed on the wastebin.

**Figure 10:**
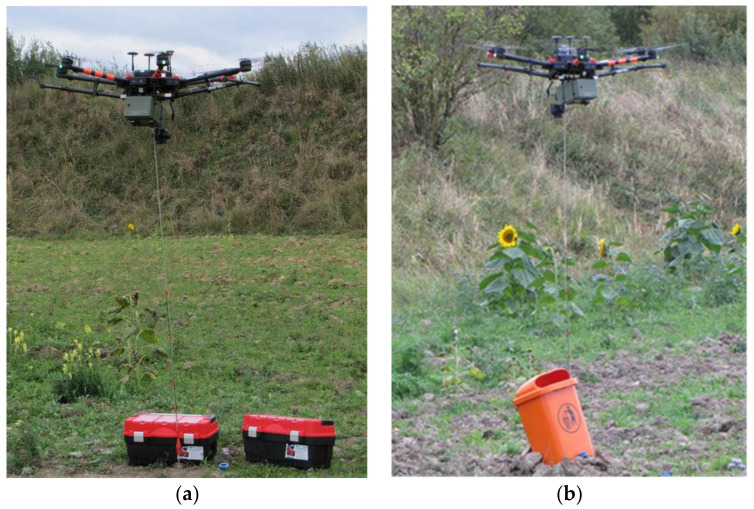
The point detection of targeted chemicals using the UAV. Sensing from: (**a**) closed cases, (**b**) wastebin.

**Figure 11:**
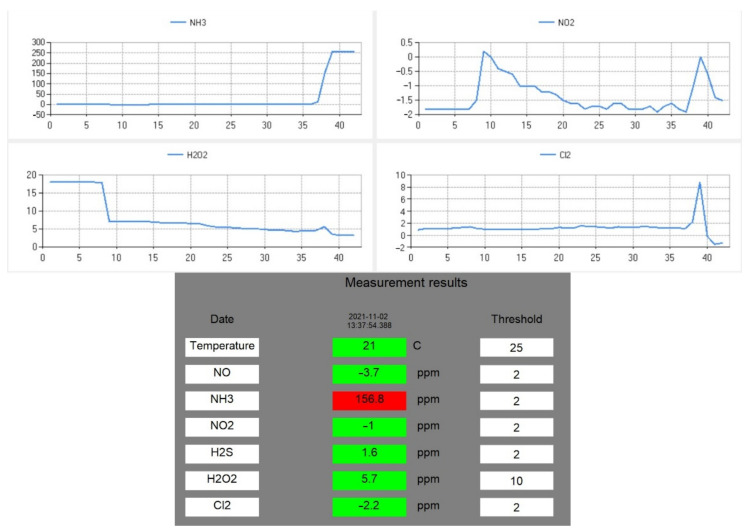
Representative sensor readings for NH_3_. The bottom screenshot shows a red indicator at exceeded threshold concentration.

To eradicate the downwash effect, a ChemDet on a UAV was equipped with long tubing. Multiple tests revealed that it is best to insert the tube into a cavity that is saturated with an analyte, which severely reduces the influence of the downwash. The tubing was 2 m long (tubing longer than 2 m was difficult to maneuver and insert into the cavity), stiffened, and equipped with a weight at its end (Figure 8). The setup facilitated handling of the UAV by the operator when trying to thread the tube into a suspicious object (Figure 10). During the tests, a possible problem of the tubing becoming entangled in the measured cavity was also recognized. Therefore, the attachment used for the tubing was replaced with a magnetic fitting representing a predetermined breaking point. This was strong enough not to lose the tubing during regular operations, but weak enough for the UAV to sever the connection with a jerky movement in case of entanglement. The screenshot from the operator panel, indicating the detection of ammonia at concentration levels that were much higher than the predefined concentration threshold, is presented in Figure 11. During measurement, the ammonia sensor reached saturation, which is visible on the graph for NH_3_ (Figure 11, top left).

## 4. Conclusions

In this paper, we presented a versatile, low-cost detector that was capable of real-time and remote sensing of up to six gases. The multi-gas sensor that was developed was tested in laboratory and field conditions. The construction allowed the sensor to be deployed on unmanned ground, as well as aerial vehicles. The long tubing allowed measurements to be taken in places that are not directly accessible to the robot or the UAV. Due to the versatile tubing, the long distance kept between the UAV and the measurement spot reduced the downwash effect. The downwash effect is not significant when a widespread gas cloud is analyzed. Depending on the kind of mission, the type of pollution measured as well as UAV construction, different sensor locations on the UAV are possible.

### 4.1. Limitations of the Study

The objective of this study was the development of a versatile, simple-to-use chemical detector that allows multi-gas sensing based on reliable electrochemical modules. Compared to the compact modules developed by commercial companies, with integrated electrochemical sensors and driver electronics that only suit their sensors, the compact modules that we used are of considerable dimensions. They are also provided with a dedicated electronic driver. Therefore, sensor replacement or adoption to the specific mission type is time consuming, and requires some screw and wire manipulation. Furthermore, the sensors we used are reliable and well documented by the manufacturer.

Another limitation of the study was sensor overload and saturation of the signal, which could last for many minutes. The effect was difficult to control, due to point detection via tubing inserted into the small cavity of a confined volume with highly concentrated vapors.

### 4.2. Future Work

The ChemDet sensor can be improved to eliminate some of the limitations. In our future work, we are going to miniaturize the sensor by using smaller electrochemical modules. They will also be implemented directly onto the electronic board, so that replacement of the sensor module will be easier. Concerning the sensor saturation, a special safety valve with an electronic control module will be used, which will close the flow through the tubing and allow the sensors to be blown with fresh air. Additional studies on the optimization of the sampling tube length are necessary to find a compromise between downwash reduction and sampling time. If required, cross-sensitivity can theoretically be accounted for in a data analysis algorithm. It could be realized by simultaneous analysis of all the sensors’ responses to certain gases. The correlation between the different sensors’ responses could help to improve the specificity [34].

Since some missions would require large area surveillance, GPS tracking should be included. Synchronized time, location, and sensor readings could provide additional visual concentration maps, allowing gas source positioning.

## Figures and Tables

**Table 1:** ChemDet parameters.

Dimensions	240 mm × 160 mm × 120 mm
Weight	1.6 kg with batteries
Power supply	Li-Ion battery pack 24 V, 2000 mAh
Power consumption	7.2 W (4–5 h of continuous work)
Remote data transmission	About 300 m
Number of gas sensor modules	6
Temperature measurement (MCP9700A)	Internal analog temperature sensor

**Table 2:** Electrochemical sensor parameters. According to the manufacturer, all the sensors present linear output, but NH_3_ < 5% full scale.

Gas Sensor	Measurement Range (ppm)	Resolution (ppm)	Maximum Overload (ppm)	Output Signal (nA/ppm)	Applied Bias VS-VR (mV)	Response Time (s)
Cl_2_	0–20	0.1	200	−900 ± 250	0	<60
NO_2_	0–20	<0.05	200	−1100 ± 300	0	<60
NO	0–25	<0.05	50	2000 ± 400	+300	<10
NH_3_	0–50	0.5	100	155 ± 30	0	<50
H_2_O_2_	0–100	<0.05	200	1100 ± 300	+300	<60
H_2_S	0–50	<0.03	100	1700 ± 300	0	<35

## Data Availability

Data available on request.
